# Community groups, organisations, and employers respond to the challenges of the Covid-19 pandemic: A story of resilience and continued vulnerability

**DOI:** 10.1186/s12889-025-22104-9

**Published:** 2025-03-06

**Authors:** Katharine Abba, Adele Ring, Peter Lloyd, Rachel Anderson de Cuevas, Shaima Hassan, Mark Goodall, Pam Clarke, Kerry Hanna, Saiqa Ahmed, Gerry Allen, Neil Joseph, Alan Price, Stephanie Tomlinson, Timothy Wilson, Farheen Yameen, Kerry Woolfall, Mark Gabbay

**Affiliations:** 1 Department of Public Health, Policy and Systems, University of Liverpool, Liverpool, UK; 2 Department of Primary Care and Mental Health, University of Liverpool, Liverpool, UK; 3 School of Health Sciences, University of Liverpool, Liverpool, UK; 4 National Institute for Health and Care Research Applied Research Collaboration, North West Coast, Liverpool, UK

**Keywords:** Covid-19, Resilience, Adaptation, Collaboration, Third sector, Voluntary sector, Volunteers, VCFSE sector

## Abstract

**Background:**

The Covid-19 pandemic profoundly disrupted societal systems, prompting community groups, voluntary organizations and employers to adapt rapidly to emerging needs. Here we present findings of a study conducted in the North West of England, exploring how groups and organisations adapted and responded to local needs at this time.

**Methods:**

We conducted semi-structured interviews with ‘key informants’ within local community voluntary, charity, faith or social enterprise (VCFSE) sector groups and organisations (*n* = 19) and large/ medium employers within any sector (*n* = 6). Interview transcripts were analysed thematically by a team of academic researchers and ‘Public Advisers’ with knowledge of local communities.

**Findings:**

Our findings reveal that community-based VCFSE groups and organisations played a critical role in addressing immediate needs such as food insecurity, isolation, and health vulnerabilities. This response was motivated by a strong sense of responsibility for the wellbeing of the clients and communities they served, and was enabled by their strong community networks, local knowledge, and ability to increase system capacity through collaboration. However, in a context of increased wellbeing needs later in the pandemic, many struggled to restart their core business, constrained by depleted resources and difficulties in interpreting and applying government ‘Covid-secure’ legislation and guidance.

**Conclusion:**

The study underscores the importance of local resilience, highlighting the VCFSE sector’s central role in addressing inequalities exacerbated by crises. It calls for substantial long-term investment to sustain this vital infrastructure, which is critical not only for recovery but also for preparedness for future societal shocks.

**Supplementary Information:**

The online version contains supplementary material available at 10.1186/s12889-025-22104-9.

## Introduction

Covid-19 was first reported in Wuhan, China in 2019. By September 2021 there had been more than 200 million confirmed cases and over 4.6 million deaths worldwide [1]. The first two cases of Covid-19 in the UK were reported on January 29th, 2020 and by March 10th, six people had died of the disease and 373 had tested positive [2]. In an attempt to slow the spread of Covid-19 in the UK, the government issued a directive on March 23rd, 2020, enforcing a period of ‘lockdown’ [3]. 

The impact of this decision was immediate [4]. All in-person services and activities deemed ‘non-essential’ were required to close. This included schools, with all but the most vulnerable children and children of ‘key workers’ considered essential to the running of the country (such as healthcare staff, postal workers, and refuse collectors) required to learn at home. People were not permitted to socialise outside their own household and could only leave home for defined ‘essential’ reasons, including work that could not be done at home, shopping for essentials, and once daily exercise [5]. Some employees who found their job role temporarily redundant were eligible for ‘furlough’, with employers applying to the Government for a grant to cover between 60–80% of workers’ wages. Despite the protective effects of the furlough scheme, there was an increase in redundancies during the first year of pandemic [6]. 

People aged over 70 years and those with certain pre-existing health issues were classed as ‘clinically vulnerable’ to Covid-19 and advised to be particularly cautious. A sub-group were classed as ‘extremely clinically vulnerable’ and advised to ‘shield’ and not leave their homes [7]. 

Through late spring to late summer 2020, as Covid-19 infection rates reduced, the most severe restrictions were lifted and ‘lockdown’ was replaced by ‘social distancing’. Social contact between people in different households was allowed within defined limits (first outdoors and then indoors), and ‘non-essential’ workplaces and public venues were gradually allowed to re-open [8, 9]. New regulations required workplaces and public venues to be made ‘Covid-secure’ [10], which involved conducting risk assessments, setting up cleaning, hand washing and hygiene procedures, supporting people to work from home, and instigating two metre social distancing where possible and where not, managing transmission risk. Additionally, face coverings on public transport [11] and in shops were made mandatory [12]. 

By the early autumn of 2020, infection rates were again rising across the UK. A second period of national lockdown started on November 5th, 2020 [13], which was eased to allow limited mixing over Christmas before a third period of lockdown was announced in early January 2021, which lasted until the early spring [14] when restrictions began to be eased again. During this time, regional variations in the level and details of the restrictions, known as ‘tiers’ were enacted in response to local variations in infection rates. Some regions spent longer than others in the higher ‘tiers’ (where restrictions were most severe), including the North West of England where this study was conducted. Most restrictions ended on July 19th, 2021, although some infection-control measures remained into 2022. A more detailed ‘timeline’ of the various restrictions has been published by the Institute for Government Analysis [15]. 

## Rationale and aims of the study

To be fully prepared to respond to the next pandemic or crisis, or indeed ongoing consequences of societal shocks such as the current cost of living crisis, we need to learn from social experiences at the level of local communities. This paper aims to contribute to the body of knowledge necessary for that learning to occur.

We undertook a study to explore how different elements of local community life were affected by and reacted to the Covid-19 pandemic in a predominantly urban area of North West England (population ≈ 1,551,800). Three work strands were chosen for investigation: individuals and households (Strand 1); voluntary, community, faith and social enterprise (VCFSE) bodies (Strand 2); and large and medium-sized employers within any sector (Strand 3).

In this paper we present findings from Strands 2 and 3, exploring how community groups, VCFSE organisations and larger employers adapted to meet the needs of their communities, clients, customers and workers as they attempted to minimise Covid-19 transmission risk, protect public health, and abide by new legislation and guidance. We describe how they responded, and we explore why they responded as they did. The findings from Strand 1 have been published separately [16]. 

In seeking to understand how and why groups and organisations responded as they did, we drew on the concepts of community and systems resilience. Although a contested construct that has been differentially defined across and within disciplines [17], the central tenet is that resilience constitutes the ability to absorb and/or adapt to disruption, stressors or shock [18]. 

In a systematic review of international studies of community resilience to ‘disaster’, Patel et al. note three general definitions: (1) ‘an ongoing process of change and adaption’, (2) ‘an ability to maintain stable functioning’ or (3) ‘a broad collection of response-related abilities’ [19]. During periods of enormous change, such as a pandemic, the first and third definitions may be most pertinent. The authors identify nine main components of community resilience: local knowledge, community networks and relationships, communication, health, governance/leadership, resources, economic investment, preparedness and, mental outlook.

In a later paper, Robertson et al. explored the concept of community resilience during workshops with a range of stakeholders [20]. The seven constituent parts of community resilience identified comprise: social ties and connections; experience and shared memory; leadership, engagement and shared responsibility; mindset, collective thinking, openness to adapt and cultural change; integration, inclusivity, equity and diversity; communications, social support and coordination; and training and exercises and identifying local need.

More recently, Suleimany et al. conducted a systematic review of community resilience in pandemic studies internationally and identified five dimensions that influenced communities’ resilience: (1) institutional, (2) social, (3) economic, (4) built environment and infrastructure, and (5) health and demographic [21]. 

In Table 1 below, we draw attention to the commonalities and differences between the constituent parts of community resilience identified in the international literature, by placing similar components alongside each other. As can be seen there is considerable overlap, although nomenclature and groupings may differ.

**Table 1:** Comparing factors comprising community resilience in the international literature

Patel et al. 2017 9 components	Robertson et al. 2021 7 key themes	Suleimany et al. 2022 5 dimensions
community networks and relationships	social ties and connections	social
local knowledge	experience and shared memory	
governance/leadership	leadership, engagement and shared responsibility	institutional
mental outlook	mindset, collective thinking, openness to adapt and cultural change	
communication	communications, social support and coordination	
health		health and demographic
resources		built environment and infrastructure
economic investment		economic
preparedness		
	integration, inclusivity, equity and diversity	

Given the recognised importance of the concept of resilience to improving public health [22] this construct is an appropriate lens through which to explore localised responses to the Covid-19 pandemic.

## Methods

### Preparation: assembling the team and toolkits

The Applied Research Collaboration North West Coast (ARC NWC) (funded through the UK National Institute for Health and Care Research [NIHR]) had access to an experienced pool of Public Advisors (PAs). The research team, comprising eight PAs and nine academic researchers, represented a range of social science and clinical backgrounds and local lived experiences. Through their interpersonal connections across local networks, three of the PAs were able to identify and introduce potential participants within the VCFSE sector. The wider PA team were able to tap into their own and their community’s experiences to provide vital context for the work, enabling coproduction of the research methods and interview topic guides.

Reporting of the co-production of this research by Public Advisors and academic staff was guided by the GRIPP2 checklist [23]. The COREQ quality checklist for qualitative research [24] was used to guide the reporting of the methods and findings of this study. To ensure that health equity issues were adequately considered, the design, analysis and reporting of the study were guided by the ARC Health Inequalities Assessment Toolkit (HIAT) [25]. 

### Study design

The aims of the study were to explore: (i) how the Covid-19 pandemic and associated restrictions impacted groups, organisations, employees, volunteers and communities; and (ii) how groups and organisations responded to the special risks and needs associated with the pandemic.

We employed qualitative research methods, conducting semi-structured interviews (some including photo elicitation [26]) with study participants. The topic guides were designed to encourage free-flowing conversation and allowed for flexibility, encouraging participants to speak about what they considered to be most pertinent. (For all topic guides see Appendix).

Participants representing local community VCFSE sector groups and organisations were invited to participate in an initial interview and a follow-up interview two months later, to explore changes in impact and response over time. Participants representing larger employers were invited to take part in a single interview, since the expectation was that key informants within larger organisations would have more limited availability to take part.^1^*

The follow-up interview used a second topic guide, with or without photo elicitation [26], depending on participant preference. The reason for using photo elicitation was to support interview discussions; this was voluntary because we felt that some participants would find it interesting or helpful and some would not.

The photo elicitation approach involved participants taking or collecting a series of photographs during the period between the first and second interview and using these as a focus for discussion. The full methods used for photo elicitation are described in an earlier paper [16]. While the main purpose of the photographs was to support the interview discussion where participants found it helpful, we present some of the photographs for illustrative purposes. We present photographic images with permission of the research participants; those containing images of people were already in the public domain, as they were used on the social media site of the participating organisation, and therefore no additional permission was sought for their use. We sought permission retrospectively, as there was no expectation prior to conducting the interviews that photographs would be published.

### Participant selection and recruitment

We used a purposive sampling approach [27] to identify ‘key informants’, defined as those involved in responding to the Covid-19 pandemic on behalf of a local community VCFSE group or organisation or large/ medium sized employer.

The PAs identified potential participants in the VCFSE group through their own community knowledge, approaching them on behalf of the research team and obtaining their permission to pass contact details to the researchers. Additional participants were recruited through a local umbrella organisation who agreed to advertise the study in their regular newsletter.

Representatives of medium and large employers were identified through local health and employer partnerships and other relevant commercial and non-commercial organisations, including ARC NWC membership lists and those of its predecessor body CLAHRC NWC (Collaboration for Leadership in Applied Health Research and Care, North West Coast).

Following a response from a potential participant, a member of the research team (KA, AR and MBG) contacted them via e-mail, including an invitation letter, copies of the study information sheet and consent form, and contact details for members of the research team. This was followed by a telephone call around a week later, when the researcher answered any questions the prospective participant might have and, if they were willing to take part, arranged a date and time for the interview that was convenient for them.

### Data collection

The interviews were conducted remotely, by telephone or video-conferencing platform according to the preference of the participant. All interviews were audio recorded and transcribed verbatim by an experienced transcriber. Initial and single interviews were conducted between July and October 2020, follow-up interviews were conducted between September and October 2020.

### Analysis and interpretation

Our analysis was thematic [28] with a coding framework being developed within NVivo 12 software [29]. Coding and theme development followed a constant comparative approach [30]. Initial codes were generated through an inductive process involving reading and re-reading of transcripts (“immersion in the data”) to identify line by line salient text that reflected participants’ thoughts, actions and experiences of the pandemic. Broader overarching themes and sub-themes were subsequently developed.

Initial reading and coding of two transcripts to support code and theme development within NVivo was conducted by seven members of the research team including research staff AR, KA, MBG and PAs PL, TW, SA and FY. Coding and theme development were further discussed during regular analysis meetings conducted using video conferencing. PAs were provided with relevant training during the study. To maintain the anonymity and confidentiality of participants, PAs who had been involved in recruiting participants (ST, NJ and GA) were not involved in the coding of Strand 2 transcripts, although they were involved later in interpreting the findings. Coding of transcripts within NVivo and further refinement of themes was undertaken by KA and AR. This was an iterative process with cycling between coding and analysis meetings.

All authors contributed to team-based reflections on the data analysis and interpretation. Because of the situation with Covid-19, all team meetings were conducted remotely by video conference.

### Profile of participants

The study included 25 participants. Of these, 19 (11 female, 8 male) represented a wide range of local community VCFSE sector groups and organisations, including community centres, children’s activities, churches, community green space, gardening and food-growing projects, food distribution projects, charity shops, and others. Six participants (3 female, 3 male) represented larger non-profit and for-profit organisations providing widely differing services to their local and, in some instances, a global marketplace; including education, research, supported living, and manufacturing and distribution. Seven participants took part in a second interview, two with photo-elicitation (2 female) and five (3 female, 2 male) without.

When presenting direct quotes, we have given participants unique identifiers beginning with their research strand (community VCFSE sector/medium or large employer), participant number (2 digits), and whether it was an initial/single interview (no indicator), a follow-up, or a follow-up with photo-elicitation.

## Findings

### Balancing risk and reward in a context of rapid change and austerity

In the following sections we explore why and how different types of groups and organisations responded to the challenges presented by the pandemic as they did, and the factors that enabled and constrained their responses. As we were only able to recruit six participants representing larger employers, and the larger employers and community VCFSE sector represented a continuum in terms of the size and formality of the organisation, we analysed and present Strands 2 and 3 together as one group.

When deciding on their responses during the pandemic, organisations had to continually assess and reassess multiple risks and benefits of their actions. For example, potential risk of exposure to and spread of infection in delivering the service versus potential physical and mental health benefits of delivering services and activities. They made informed and nuanced decisions in response to changing community needs, regional and national laws and guidelines, and shifts in the nature of the pandemic. There was no central strategy to guide them. These choices presented moral and ethical dilemmas, often without an optimal solution, tied to a huge sense of obligation and responsibility on the part of decision-makers.

*There’s kind of like a moral dilemma in a lot of organisations in that… there is that moral obligation to look after your staff and volunteers as well but it’s also giving the people who need your services and support.* [community VCFSE sector _01]

In the face of these moral dilemmas and risks, organisations also had to consider their costs, revenue, and longer-term viability. Many reported having started the pandemic with reduced financial viability due to a decade-long government austerity programme.

*Even before the pandemic there’s a lot of cost cutting here*,* there and everywhere… and what gets affected in the end is your social care and your adult social care because they haven’t got the funding there.* [community VCFSE sector _25]

### Options for local organisations: Close or carry on and adapt

Participants described four broad categories of response to the risks and conditions associated with the pandemic: (1) closing local operations; (2) moving to remote working or remote delivery; (3) implementing safety adaptations to enable in-person activities; and (4) pivoting services to address essential needs of the community. Organisations often adopted more than one response and continually revised their approach as infection rates, scientific knowledge and political responses changed over time.

Many organisations involved in the local retail and service economy (charity shops, hairdressers, and some community centres, cafes and sports facilities) had to close completely, intending to re-open when legislation allowed and they assessed risk levels to be acceptable.

Some services and activities that had previously been provided in-person were quickly adapted to remote delivery (telephone, home delivery or online). This included education, church services, advice, and some social and leisure activities. The offer was often developed and expanded over the course of the pandemic, as staff and volunteers learned new digital skills and developed new resources. Where organisations could remain operational with staff and volunteers working remotely, dilemmas of balancing risks with providing services were avoided.

*We had to make a virtual adaptation… what we have been doing during through Covid is a telephone support service… from there we started delivering one-to-one telephone courses*,* and then I initiated on behalf of the charity a digital series (of mental health and wellbeing interventions) as well.* [community VCFSE sector_34]

**Fig. 1:**
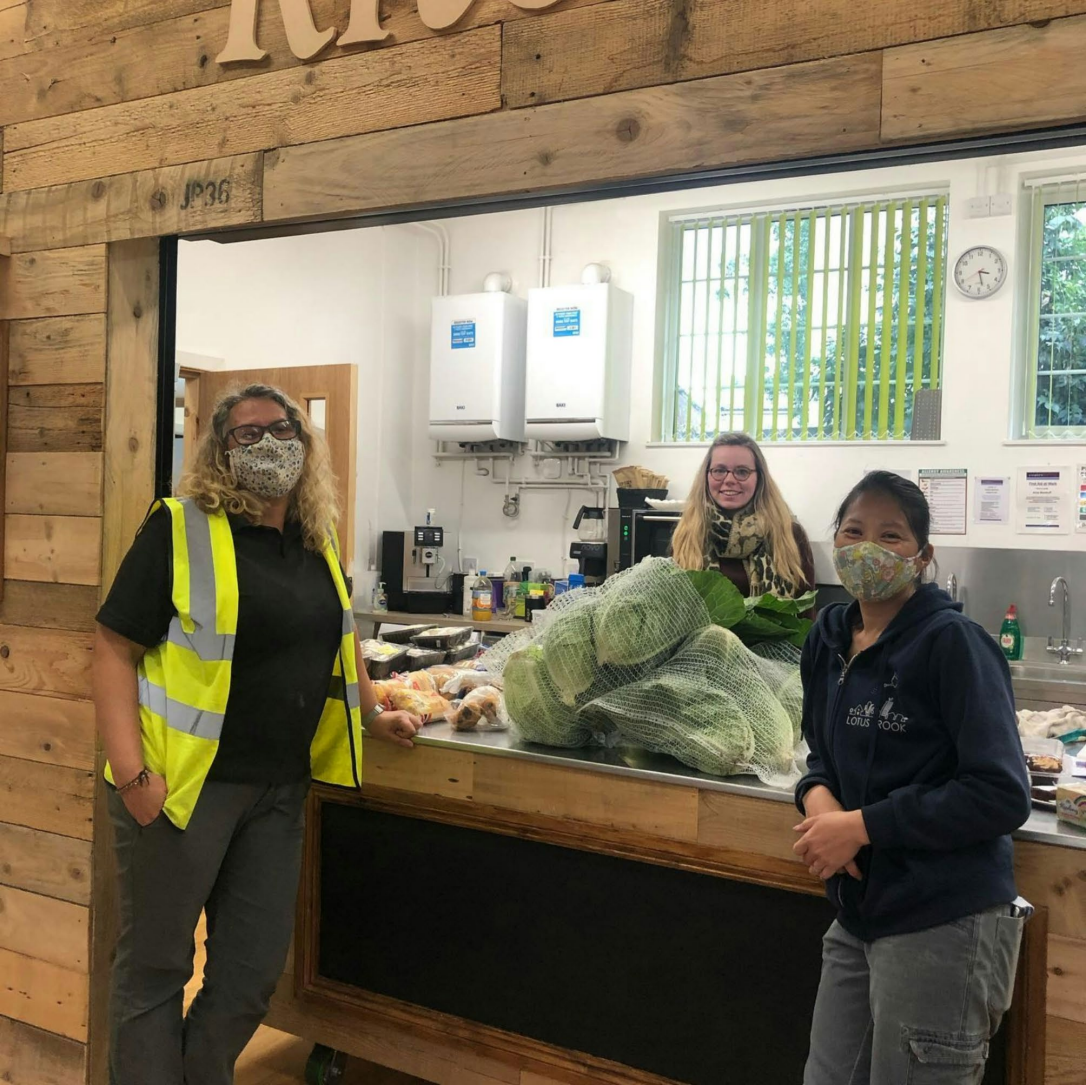
Food production and distribution continued throughout the pandemic [S2028_PE]

**Fig. 2:**
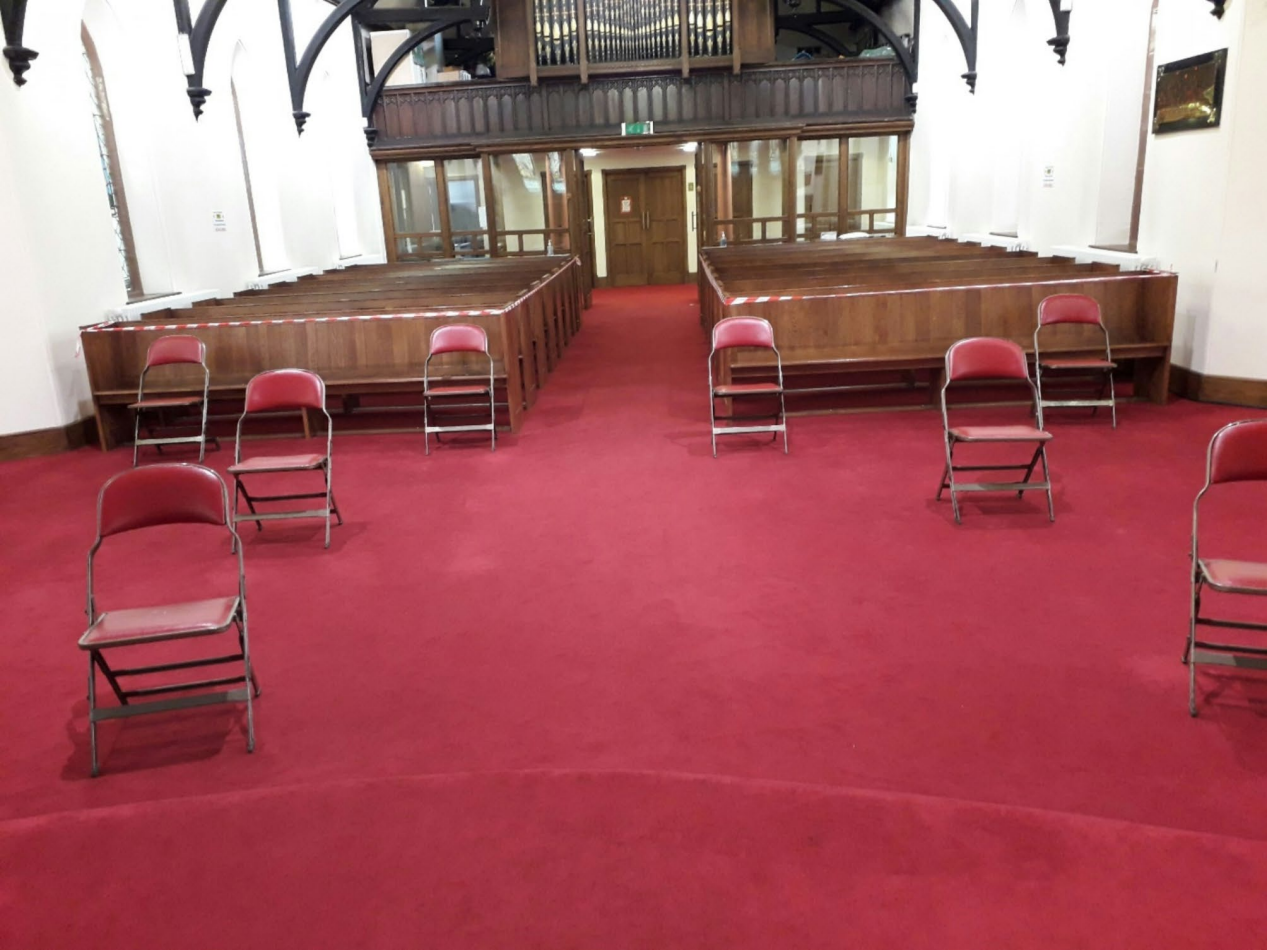
A church made ‘COVID-secure’ ready to re-open - additional photographs showed the small congregation all wearing face-coverings [S2_26_PE]

Some organisations, including food organisations, care and housing providers, schools, and those delivering commercial goods, continued to provide essential or permitted in-person services throughout the pandemic, implementing safety adaptations to reduce infection risk. Others, including churches, community centres and adult education providers, adapted, or were planning to adapt, buildings and services to enable them to re-start in-person activities once the risk levels and legislative framework allowed. Often these adapted services were limited in the numbers of people they could accommodate, and the quality of social interactions were different, as physical distancing and face-covering created barriers to natural communication. Figures 1 and 2 illustrate the safety adaptations made by a food distribution charity and a church. 

The process of making venues ‘Covid-secure’ was complex and often resource-intensive, involving the reading and interpretation of government guidelines, weighing up different risks and priorities, planning, purchasing new equipment, training and disseminating information, and re-organising spaces and activities. This placed a significant burden on organisations and the staff and volunteers working in them, in some cases making it difficult to re-open. We expand on this later.

In the framework of Maslow’s hierarchy of needs [31], many VCFSE organisations initially shifted their focus to meeting basic needs, including food aid for households facing hardship; telephone calls to people who might be isolated or vulnerable; and delivering essentials such as food and medicine to those unable to do their own shopping. Often these activities were operational within a few days of lockdown being announced and could be undertaken with minimal risk of Covid-19 transmission. Such activities and were often considered ‘essential’ and therefore permitted within Covid-specific legislation, even during periods of strict ‘lockdown’.

*They’ve been able to offer food outside… Instead of having it in the centre*,* they’ve set up like a little market stall just by the door and people have come and got the food from there.* [community VCFSE sector_39]

However, committing resources to these activities could reduce capacity to deliver usual services, or risk organisations’ future viability.

*My mind’s whirring about what I can do*,* because obviously I’ve got to balance between giving them some money from the [Club 1] but keeping the [Club 1] running… and kind of*,* you know*,* helping out our local community and children… it’s a difficult one.* [S2_04_FU}

### Factors enabling or limiting adaptation

We identified several factors, independent of infection risk, that positively and negatively affected groups and organisations’ ability to continue to support customers, clients or communities during the pandemic. These factors applied mainly (but not exclusively) to the VCFSE sector, and their ability to pivot or expand services to meet the immediate essential needs of their communities. They also affected re-opening of venues and in-person activities once infection risk was reduced and the legislative framework allowed. We describe these factors below.

### The importance of embeddedness in the community

Groups and organisations that were highly embedded in the community were able to respond quickly and flexibly due to their detailed local knowledge of their community and their connection to local networks (including pools of volunteers) which ensured that resources were effectively distributed to where they were most needed.

*We were very lucky having these local groups who were offering meal/medication deliveries and teams within our organisation who had gone from being*,* you know*,* asbestos and gas specialists to become a team where they were willing to go and do a bit of shopping and deliver it to our older customers. [Medium/large employer _04]*

Participants rated local community responses favourably above local government responses, which ranked higher than national responses in turn, emphasising the importance of local knowledge and infrastructure, and the flexibility of local staff and volunteers at this time of crisis.

*Our community hubs and groups*,* the groups that they live and know and have grown up in the area and they know the vulnerable ones… The Council has done some kind of support system*,* but it’s not gone as deep as these organisations have. And they’ve been round*,* you know checking on pensioners*,* they’ve been going and delivering food parcels.* [community VCFSE sector_39]

*The NHS responders was a fine idea…but actually we*,* each local authority had their own bank of volunteers that they’d recruited 6 weeks previously.… NHS Trust came to us and say “we need 57 volunteers to deliver prescriptions” - we had people there ready to go.* [community VCFSE sector_01]

### Connections between groups as a key enabler

The local response was further enhanced by pre-existing and newly developed communication networks and service delivery partnerships between mainly VCFSE groups and organisations. For example, food banks collaborated with local community groups to deliver food to people in need. In some areas, local authorities organised regular meetings of organisations involved in the Covid-19 response, helping them to identify gaps in provision and enabling a more coordinated response across a wider area.

*We connected up with community groups who were already operating in the community and said “right guys this is what we can offer… (food supplies)”… a collaborative effort… Four hundred lunches being delivered out every single day last week… some of those were given out through [name of organisation] and shops where they know that the kids will come and hang out and they haven’t got any food.* [community VCFSE sector_12_follow-up]

*Through the Council…we all sort of get together……We share news*,* we share best practice*,* we share thoughts and feelings……that’s helped me sort of figure who’s doing what*,* where they’re doing it and maybe where the gaps are.* [community VCFSE sector_01]

### Access to emergency funding

Securing sufficient funding circumvented the dilemma of responding to immediate essential needs versus remaining viable and able to provide usual activities. During the early months of the pandemic, emergency funding was available from various sources, and participants described both successful and unsuccessful attempts to access these funds.

*That kind of lasted about 6 weeks with the funds that we had*,* but we didn’t want to stop it*,* so I just engaged with one of our local councillors here who told us there was some funding available to for things exactly like this*,* and I think they then funded that for the next kind of two and a half, three months*. [community VCFSE sector_04_follow-up]

*As our meals were scaling up*,* we ran out of storage space so we needed a small freezer unit… we went to our local town council and asked for a £500 grant which they can award. They made us wait 6 weeks… (and) they only gave us 400.* [community VCFSE sector_27]

### The need for clear, consistent, and readily available legal and public health guidance

Organisations providing essential in-person services, or re-opening public venues or in-person activities that had initially been required to close, were required to adhere to Covid-specific laws and government guidance on infection control, which the UK government referred to as making workplaces ‘Covid-secure’.

Central government guidance was experienced as long, complicated, open to interpretation, and frequently changing. Participants reported that new versions of guidelines did not highlight changes, making it necessary to read documents in their entirety. The workload involved in keeping updated could feel overwhelming, especially during periods of rapid change, and the uncertainty contributing to a sense of anxiety that could be paralysing. For example, some participants felt the lack of clarity in the guidelines potentially left them open to legal action.

*… there’s so many changes on a daily basis*,* not only in legal documentation that could change maybe every 2 weeks… but the nature of your building*,* it could be that one member of staff has got symptoms… It’s constant.* [medium/large employer_03]

*I try and work within the*,* you know*,* the never-ending stream of new rules and new regulations and new whatever and… I feel I’m sort of saving myself from drowning rather than being effective and being helpful to other people.* [community VCFSE sector_33_follow-up]

*One of the things that keeps coming up is the issue of insurance and how [community centres] cannot re-open because they’re so worried that they might get sued or they might have an issue with [Covid]…having to be shut down straight away* [community VCFSE sector_12].

Once the guidance was read, digested, and a risk-assessment undertaken, the process of planning and implementing the necessary safety adaptations created further work for staff and volunteers and could incur substantial costs.

*We could have (legally) opened our [venues] at the end of July/beginning of August*,* but we haven’t because we’ve had to do all the planning…we’ve had to get sanitisers fitted*,* notices up*,* all furniture moved away*,* everything packed away. All [hand-held equipment] that we normally use*,* we’ve had to pack all those into boxes. Some of us feel as though we’ve lived down there.* [community VCFSE sector_26]

Participants representing non-profit community venues perceived the rules and guidance to give them a low priority in discussions about re-opening compared commercial and public sector employers; for example, pubs were allowed to open before community centres. Community centres also had the added complication of hosting multiple groups and activities, each governed by different rules and subject to different risk assessments.

*Community centres [guidance] come out a bit piecemeal… we were left to the very end before we could even look at getting back to normal because multiple organisations are based here… that’s why we’ve only got our first groups coming in now*,* but pubs have been open for weeks.* [community VCFSE sector_40]

The challenges of interpreting guidelines were most acute for smaller local VCFSE groups and organisations without in-house health and safety teams. However, for some, support was available through a larger affiliated organisation (for example a sports or church body) that translated government guidelines into ‘user-friendly’ guidance for their specific setting. This helped to reduce the burden on small groups.

*There’s the likes of NCVO [The National Council for Voluntary Organisations]*,* they got the government guidelines and they made it a lot more user friendly…. and that seemed to work a heck of a lot better than trying to figure out what the Government was saying*. [community VCFSE sector_01]

### The challenges ahead: Increased community needs and depleted organisational resources with worsening austerity

During the study period, participants observed an increase in mental distress (in children as well as adults) and financial distress (with associated food insecurity) within their communities, client groups and staff. They expected those needs to continue post-pandemic; some organisations had already adapted services to respond to these new needs.

*It’s not just stress and anxiety as a result of the pandemic*,* it’s financial insecurity*,* access to healthy food and isolation… it’s all the things that we’ve been trying to tackle anyway are now just like massively exacerbated…* [community VCFSE _28_photo-elicitation].

*We have now initiated a new younger women’s project*,* which is not something that we normally do… we work with 18 and over. But we’ve released a new project for young women aged 14 to 17*,*… because we know the impact on young people’s mental health is going to be severe.* [community VCFSE sector_34]

At the same time, organisations were experiencing a depletion in their resources that left some in a precarious financial position. Many had increased expenditure to make premises Covid-secure or address essential needs within their communities. Others had experienced a fall in income, as many commercial and fund-raising activities could not take place, and funding bodies concentrated on the immediate crisis response to the pandemic. Some experienced both increased expenditure and decreased income.

*Financially it’s going to be a challenge to the [business] especially given what we’ve invested to keep people safe*,* you know*,* in time and resources.* [medium/large employer_05]

*Obviously*,* we’re not getting the funds in that we did previously*,* you know the money that we’re getting in isn’t even covering the bills.* [community VCFSE sector_04]

Some participants were concerned that employees and volunteers were in danger of becoming ‘burnt out’ due to high workloads associated with implementing adaptations and meeting additional needs. Some had lost touch with volunteers (mainly those who were older or more vulnerable), leading to concerns that their human resources were also becoming depleted.

*…We’ve spent millions and millions of pounds to get the [workplace] ready for people and that’s taken a lot of time and planning and effort and that’s*,* you know*,* taking its toll on a lot of people. You know a lot of people have not had many holidays this year… and that’s not healthy for people.* [medium/large employer_05]

Participants representing VCFSE sector organisations highlighted the need for more long-term investment in the sector if services were to survive and support increasing need for services into the future. The also suggested that increased direct financial support to individuals and families, and improved public services (especially improved access to mental health support) would reduce the need for voluntary sector support.

*There’s plenty of people willing to do the work and to support people but without the capacity and without the funding it’s not going to happen… I can’t really separate it out or make it any clearer*,* there is not enough money coming in to support families*,* to support community groups*,* to support the third sector*,* there just isn’t.* [community VCFSE sector_12_follow-up]

## Generating ongoing support to build new ways of working

### Combining remote and in-person service delivery

At the start of the pandemic, many organisations had, out of necessity, adapted services that were previously delivered in-person to a remote delivery model, many of which were perceived to have worked well. Over time, organisations and service-users had acquired new technology and skills, enabling them to participate on-line. Online digital activities could sometimes reach a wider audience than in-person activities, including some previously excluded groups (e.g. those who were housebound), and encouraged the participation of young people. The use of remote communication technology also reduced travelling time and expenses associated with business meetings, thereby reducing organisational outgoings and improving job efficiency and work-life balance for some.

*We had more people attending (annual meeting) than we have ever had when we’ve done it live… it was fine*,* we discovered you can do voting on it… (and) some people have really*,* really enjoyed doing the Zoom services*,* being able to attend church*,* if you like*,* from home*,* some of them might not normally be able to come.* [community VCFSE sector_26_follow-up]

*We would probably be in [name of city] once or twice a week and I think we’ve all looked now and think ‘why did we do that?’ You know*,* we can do a [name of city] meeting on Teams or Zoom and get the same result…* [medium/large employer_02].

However, participants identified that some members of their communities remained ‘digitally excluded’ with no or limited online access, due to financial constraints or lack of confidence in using digital technology. This was recognised as exacerbating existing inequalities, with groups that were already disadvantaged before the pandemic being most likely to be excluded. People with disabilities such as dementia or learning disabilities often found it difficult or impossible to engage with online activities, even if they technically had access to them. Remote activities also were noted in many cases to be an inferior substitute for their in-person equivalents, as for many people and activities it was less effective, less pleasurable, or had reduced functionality.

*It’s removed all of the safety nets*,* so the spaces where parents could go*,* the spaces where older people could go*,* the libraries… there is no safety net now in there for people to get any support… if you can’t afford food*,* you can’t afford WIFI either.* [community VCFSE sector_12_follow-up]

*Our offer for the elderly… a lot of them haven’t got computers and phones even*,* that’s why they like just walking down the road*,* we are right in the centre of the community here and they can*,* you know*,* engage with people.* [community VCFSE sector_27]

Given the advantages and disadvantages of remote versus in-person provision, many organisations hoped to provide both options in the future, although this would be constrained by the availability of resources.

*We are a very busy centre under normal circumstances and obviously our kind of frontline staff they are occupied already… so it will be limited what I can do with the digital series*,* but I do very much hope to continue it*,* even if it’s on a lesser level…similar with the telephone support service…* [community VCFSE sector_34].

## Creating new partnerships and collaborations

The pandemic response had prompted closer joint working across agencies. VCFSE sector groups and organisations hoped to be retain and build on these new working relationships to help better address anticipated increases in mental health and financial needs within their community.

*Food that we’ve grown…the majority of it has gone to [foodbank]…and then some have gone to like a couple of community centres as well… we worked in this way because of Covid and we will continue to work in this way.* [community VCFSE sector_37]

*Trying to think ahead to… how we can support… wellbeing all round… we need to find out more about what’s on offer*,* maybe more connections just in terms of referral processes and things like that… part of where we see we fit is that little bit of a bridge to introducing people to mainstream support and being a connector.* [community VCFSE sector_28_photo-elicitation]

## Discussion

The Covid-19 pandemic was a ‘shock’ to communities and organisations, starting as a public health shock and quickly developing into a broad-spectrum health, social and financial crisis. The ability of communities to respond to, cope with, and recover from these shocks depends on factors determining their ‘resilience’, many of which were apparent in our study.

We found that local community-based groups and organisations were crucial in spearheading the early crisis response, supporting the most disadvantaged within their local communities, particularly those experiencing food insecurity or isolation. This aligns with findings of research conducted in other areas of the UK at the time [33–40] and in other contexts, including the aftermath of extreme weather events in the USA [41, 42] suggesting this phenomenon is not specific either to pandemics or to the UK.

Our study was conducted during a period of turbulence and uncertainty, experienced by our participants as a climate of anxiety and challenge, but also of creativity, as new ways of working were developed. Such adaptiveness has been identified previously as important for community resilience in the context of shocks [19, 20]. 

Bene et al. conceptualised resilience as comprising three types of capacity: ‘absorptive’, ‘adaptive’ and ‘transformative’ [43]. Within this model, during times of stress, communities or systems may remain unchanged and continue to function (absorptive capacity). When absorptive capacity is exceeded, adjustments are made to maintain function without ‘qualitative’ change to the system (adaptive capacity). Should this adaptive capacity become overwhelmed, communities or systems must make more transformative changes (transformative capacity) to adequately respond to effects of the shock. As groups and organisations responded to local needs during the pandemic, we saw examples of resilience through ‘absorption’ (meeting increased needs and expenditure through the additional efforts of staff and volunteers and using financial reserves or applying for emergency funding), ‘adaptation’ (the shift to remote working and service-provision) and rapid ‘transformation’ (providing completely different services in response to changed circumstances).

This process of adjustment and innovation was ongoing and responsive to changing local needs and conditions, a finding in common with other studies conducted with the VCFSE sector at this time [44]. Embeddedness within the local community, a feature of many of the VCFSE sector organisations in our study (as well as some in the public and private sectors), was crucial in enabling this transformation. A key advantage of this embeddedness was the detailed local knowledge of their communities, which enabled them quickly to target resources where they were most needed. This type of knowledge, along with the relational skills of voluntary sector organisations, has been identified previously as critical to a rapid response [35, 45]. The inter-connectedness of local organisations supported the development of new connections and networks that increased the scale of the response. Previous studies have described how organisations’ resilience to shocks was contingent on their ability to leverage such networks [32, 44, 46], and highlight [19, 32] the importance of building on such connections in supporting local recovery efforts.

Embeddedness and connectedness were also important for mobilising volunteers, further increasing response capacity. This reflects national findings that around 3.4% of the UK population volunteered specifically in response to the pandemic [47] and highlights the importance of ‘social solidarity’ arising from common purpose and understanding between community members and organisations in community resilience to pandemics [21]. 

Embeddedness within the community, relationships between different groups and organisations, and social solidarity can also be understood in terms of social capital, an important component of community resilience to shock [45, 48]. Of particular relevance is the concept of *bridging social capital*, which encompasses connections between people who share similar socio-economic conditions, perspectives, perceptions and hardships [49, 50], and can extend to local groups and organisations with established bonds within communities. We also saw examples of *linking social capital*, where established relationships with external organisations enabled access to resources, including emergency funding, outside of the immediate community [41, 45]. 

Our study also offers insight into *why* VCFSE sector groups and organisations responded as they did. Vital here was the sense of responsibility of decision makers to their staff, volunteers, client group and wider community, and the responsibility carried by volunteers to the communities in which they often lived. Participants’ narratives described how they continually wrestled with the responsibility of providing services whilst minimising the risk of transmitting Covid-19, especially to groups perceived as more vulnerable. This illustrated how important the set of values held by key players is to the creative resolution of a broad-spectrum crisis such as a pandemic.

The limited data we have from public sector and other employers is also about adapting to the new realities, perhaps motivated more by the needs to continue to fulfil contracts and deliver services. This process has previously been characterised as ‘disaster entrepreneurship’ to enable business continuity [51]. There was a parallel process of adapting central government guidance to local realities and needs, reflecting comparable challenges and priorities [52]. 

While we identified many strengths within communities and voluntary organisations during the pandemic, we also identified vulnerabilities that may have hindered resilience. Pandemic policy in the UK was conducted by regulatory decree from central government while the practical applications of these requirements were worked out by actors in local spaces. This created enormous challenges for many groups and organisations in our study, who sought to grasp what was required of them by law, interpret what that would involve for them in practice, and then negotiate this locally with clients and workers.

The UK Covid-19 Inquiry [55], set up to examine the UK’s response to and impact of the Covid-19 pandemic, highlighted some serious flaws at central government level that meant the UK ‘lacked resilience’ in the face of the pandemic. These included structural issues within UK government departments, inflexibility of pre-prepared pandemic plans, issues that affected decision-making, and failure to take adequate account of existing health and social inequalities. A central government in turmoil presented those working at local level with conflicting options and expectations.

These challenges often constrained the ability of local organisations to meet the ongoing health and wellbeing needs of their clients and communities. For example, community centres, which provide vulnerable members of the community with vital opportunities to meet with others and engage in meaningful activities, were often unable to re-open long after they felt it was safe to do so. Inadequacies in central government guidance required local actors to use their own initiative to make things work effectively; for some, this seemed indicative of a low priority given to non-profit community resources. These factors may have reduced the capacity of VCFSE to respond to local need, especially during the recovery phase. This supports characterisation of resilience as a multilevel construct with influences across levels [53] and recognises that local responses to shocks are influenced by responses at different levels of the wider system [54]. 

It is established that existing conditions can amplify or dampen the shock effect of pandemics [54]. Our participants reported pre-pandemic trends of increasing population need and reductions in public services, and the Covid-19 inquiry [55] highlighted that public health and healthcare responses were ‘constrained by their funding’. This left the voluntary sector as first responders with depleted resources. Whilst the local VCFSE organisations in our study demonstrated remarkable resilience, many were concerned for future, as the pandemic depleted their resources further and exacerbated health and financial inequalities and needs.

Other UK research found that the pandemic negatively impacted the financial resilience [56], of both individuals [57] and local authorities [58]. In our study and others [35], local authorities often played an important linking role in the VCFSE pandemic response, for example in facilitating access to emergency funding or coordinating local responses. The future capacity of the VCFSE sector to respond to shock may depend not only on their own financial resilience, but that of the communities they serve and on larger structures, such as local authorities, that might support their response.

Despite these challenges, the VCFSE sector was able to increase its intrinsic capacity during the pandemic, by developing wider partnerships and harnessing the potential of digital technologies. The centrality of the VCFSE sector to the relief effort may be in danger of being overlooked as the pandemic gives way to the cost-of-living crisis, and tight public sector budgets. 

Our finding that the shift to remote working expanded the potential for some previously excluded people to get involved was also identified in research investigating the impact of the pandemic on public involvement. However, inclusive participation will require organisations to support the costs of IT equipment, internet connectivity and training [52] as well as continuing with ‘in-person’ services where these meet people’s needs better.

The pandemic exposed and exacerbated existing health and social inequalities; with those already living in vulnerable circumstances most affected [59–61]. Such communities have been described as experiencing a ‘syndemic’ of Covid-19, chronic illness and poverty [62]. Before the pandemic, levels of income inequality in the UK had already increased over the previous four decades [63]. The UK remains a country of substantial health inequalities [64] with high levels of poverty, particularly among children [65], adding to pressures on the national health and care system. Consequently, the most disadvantaged communities will continue to require targeted financial, social, emotional, and mental health support. The VCFSE sector is central to the provision of these types of support. However, the sector was already stretched before the pandemic arrived, and the pandemic made the situation worse [66, 67]. Mahase [67] found charities anticipated 12.4 billion loss of income due to the pandemic, and recent reports suggest that charities are currently finding themselves on the verge of bankruptcy [68]. Some of the organisations included in our study were so concerned about depletion of their financial resources that they questioned their ability to respond to any future crises. A need was identified for increased long-term funding for families, communities and for the VCFSE sector if needs are to be met.

### Strengths and weaknesses of study

A major strength of this study was the multidisciplinary team involved, including the Public Advisors, who enabled us to identify appropriate participants and provided valuable insights into the lived experience of community members.

A potential weakness was the self-selecting sample, which may not be fully representative the experience and views of the community-based VCFSE sector and medium/larger employers in the study area. However, our sampling methods enabled the inclusion of organisations that had been able to adapt and respond to the situations created by pandemic and provided insight on how that was possible. Many larger commercial and statutory employers were unable to take part largely because they had already received many requests for information and were unable to take on another.

The predominance of local VCFSE sector voices over larger employer voices was unintentional and arose through difficulties in recruiting representatives of larger employers. We were unable, as initially planned, to analyse and present the findings for different types of organisations separately, and instead pooled our data for joint analysis. We attempted to make the best of the data available by concentrating mainly on the experience of the VCFSE sector, using the additional data to identify areas of commonality and difference across different types of and size of organisation.

### The use of photo-elicitation

Only two participants chose to take part in a follow-up interview with photo-elicitation, perhaps because of the additional time commitment involved. We found these two interviews focussed more on emotions than the other interviews. Participants vividly described the pleasure of meeting colleagues in-person, outdoors, after months of meeting only remotely, and of being able to celebrate an elderly parishioner’s birthday with one person visiting her in-person while a choir sang for her on video call. Photographs illustrated the slightly desolate atmosphere of a physically distanced church service, where members of the congregation wore face-coverings and were not allowed to socialise afterwards. Interviews using photo-elicitation also described practical issues, such as the work involved in adapting spaces, in at least as much detail as follow-up interviews without photo-elicitation. From these small numbers, we cannot conclude whether this difference was due to the methods itself, or to the interests and preferences of the participants who chose photo-elicitation.

## Conclusions

Community-based voluntary services were vital in the early months of the pandemic [32, 33, 39] and continue to be so, with the crisis having shifted to the cost-of-living crisis. Once again, the greatest burden is falling on the most disadvantaged and on already challenged local communities. Post-pandemic, many of the organisations best placed to help are nearing breaking point. Without substantial long-term investment [4, 32] we are in danger of losing a resource and infrastructure that proved, when Covid-19 hit, to be an essential front line emergency service [66, 67]. 

As the UK Covid-19 Inquiry [55] starts to publish its findings, this seems an appropriate time to celebrate the critical role played by the VCSFE sector. Crisis management through central and regional statutory organisations fell far short of what our participants reported was needed; actions set in the local social context are also vital. As we have shown, the flexibility and creativity of the VCSFE sector can be vital for achieving effective outcomes, especially in areas of deprivation. Allowing the sector to atrophy through lack of funding can weaken its ability to play an essential role once a crisis arrives. Central choices to cut local authority funding year-on-year have collateral effects not just on the delivery of statutory services but also on the delicate but vital structures and networks of the VCFSE sector. This paper has provided evidence for how vital they are.

In planning to respond more effectively to future pandemics and other ‘shocks’ to communities, policy needs to recognise the importance of the sphere of local governance in general. The local authority role is critical, as is the VCSFE sector. From the evidence set out here, it would be a travesty to allow both to have to confront another such crisis without the voice, influence and appropriate resources to respond properly. In the meantime, it is essential to recognise and then to properly resource the contribution of the VCSFE sector in general so the sector’s capacity can be maintained as a hedge against future crises. The story told here is supported by other research conducted in the UK during the pandemic, and we expect it will have been replicated up and down the country.

## Electronic supplementary material

Below is the link to the electronic supplementary material.

Supplementary Material 1

Supplementary Material 2

## Data Availability

No datasets were generated or analysed during the current study.

## Footnotes

1 * In the event, recruiting larger employers still proved very challenging despite a wide variety of approaches through different routes - the managers explaining they were overwhelmed with requests for information and data on top of their pandemic management workloads.
