# Supplementary material for: Community groups, organisations, and employers respond to the challenges of the Covid-19 pandemic: A story of resilience and continued vulnerability
Source: BMC Public Health. 2025 Mar 6;25:890. doi: 10.1186/s12889-025-22104-9 (PMC11884177; doi:10.1186/s12889-025-22104-9)
Supplement: Supplementary file 1 — Supplementary Material 1 [file 12889_2025_22104_MOESM1_ESM.docx]

**Topic Guide: Communities (Strand 2):**

**Perceptions of risk and experience of the COVID-19 pandemic for households, communities and organisations in the Liverpool City Region (COVID – LIV Area B UK)**

**Communities Topic Guide**

- Introductions/welcome//thanks
- Check whether the participant has any questions prior to starting the interview. The interview will last approximately 60 minutes
- Advise that audio recording is being switched on
- Review consent form (which they will have returned via e-mail?) – reiterating right to withdraw, confidentiality and anonymity etc.
- Check still happy to participate – verbal consent:
  - Participant to state their name
  - Participant to state they have read through the information sheet and know what the study involves
  - Participant to state that they agree to take part
  - Where participant is providing verbal consent only, researcher requests permission to switch on audio recording and verbal consent form and completes for (name of participant, date of verbal consent, researcher adds their name and date.

**Introduction**

We are seeking to understand better what people think about the impacts of the COVID-19 pandemic on communities and the ongoing risks it poses to communities. We also want to understand more about how communities are experiencing current advice and restrictions requiring them to socially distance from others in their community, and how community groups are responding to those experiences.

*Could you start by telling me a little bit about yourself and your role in the community?*

**Risk**

*Now I’ll ask you a few questions about the impacts and potential impacts that you see the coronavirus pandemic is having, or think there is a risk of it having, on your community (or community group).*

***What do you think are the main risks to the local community from COVID-19/coronavirus?***

*Additional prompts:*

- Risks of people becoming infected, and reasons
- Potential consequences of:
- People becoming infected
- The ‘social distancing’ and isolation measures brought in to control the pandemic

*Thinking now about the advice to self-isolate if you or a member of your household has symptoms of coronavirus:*

- What do you think of this advice on self-isolation for your community?

*Additional prompts:*

- Particular challenges within the community regarding self-isolation
- Anything that has helped members of the community to self-isolate

***Now, thinking about the government advice about social distancing to reduce the risk of COVID19, what do you think about this advice?***

*Additional prompts:*

- Easy or not for the community to follow the social distancing directives and advice
- Any changes as the advice has changed
- Anything that has made it difficult for people in your community to stay at home
- Anything that made social distancing easier for community

**Impact**

***Now, thinking about any impacts of government advice and restrictions within the community:***

What impacts have you noticed the restrictions having within the community?

How have the community been responding to the restrictions? (e.g. practical responses)

*Prompts;*

- New ways of working (details)
- Personal/community group involvement (explore)

***In general, what do you think about the coronavirus information that has been coming out from UK government?***

How might this information have been improved?

*Prompts:*

- to better inform people about risk
- to help people understand and comply with government directives

***Do you have any concerns about longer-term consequences and changes after the pandemic?***

***Thinking about the future, what do you think the key priorities will be for the community to recover from the impacts of COVID19 and why?***

And finally, is there anything that I haven’t asked about that you would like to add before we end the interview?

 Thank you for participating.
