# Supplementary material for: Community groups, organisations, and employers respond to the challenges of the Covid-19 pandemic: A story of resilience and continued vulnerability
Source: BMC Public Health. 2025 Mar 6;25:890. doi: 10.1186/s12889-025-22104-9 (PMC11884177; doi:10.1186/s12889-025-22104-9)
Supplement: Supplementary file 2 — Supplementary Material 2 [file 12889_2025_22104_MOESM2_ESM.docx]

**Topic Guide: Organisations (Strand 3)**

**Perceptions of risk and experience of the COVID-19 pandemic for households, communities and organisations in the Liverpool City Region (COVID – LIV B UK)**

**Organisations Topic Guide**

- Introductions/welcome/thanks
- Check whether the participant has any questions prior to starting the interview. The interview will last approximately 60 minutes
- Advise that audio recording is being switched on
- Review consent form (which they will have returned via e-mail) – reiterating right to withdraw, confidentiality and anonymity etc.
- Check still happy to participate – verbal consent:
- Participant to state their name
- Participant to state they have read through the information sheet and know what the study involves
- Participant to state that they agree to take part
- Where participant is providing verbal consent only, researcher requests permission to switch on audio recording and reads out verbal consent form and completes for (name of participant, date of verbal consent, researcher adds their name and date.

**Introduction**

We are seeking to understand better how key organisations think about and manage risk associated with the COVID-19 pandemic for their employees, the people they serve or do business with, and the wider community. We are also interested in how COVID19 and the associated restrictions are impacting on the organisation and the communities they serve.

Could you start by telling me a little bit about yourself and your role in <insert organisation here>.

**Risk**

***What do you think are the main risks to staff of [organisation name] from COVID19/ coronavirus?***

*Additional prompts:*

- Risk of them contracting the virus (explore reasoning)
- Risk associated with the virus
  - Personal risks for employees
  - Risks to the organisation and the work it does
- Changes since lockdown in March

***What do you think the risks are to your clients/customers/residents etc (as appropriate if relevant) from COVID19/corona?***

*Additional Prompt:*

- - Risks of transmitting the virus to customers/clients or the wider community

**Health protection advice and measures**

*Now, thinking about current health protection advice and directives around coronavirus:*

***What directives or advice, if any, have you had about health protection measures you and your employees should be taking to protect themselves and others from infection?***

*Additional prompts*

- Sources of advice (explore: where, what, when and any change)
- Thoughts about advice (easy or not to understand; implementation e.g. social distancing at work, home working, PPE access, access to testing)
- How might this information have been improved to better inform organisations?
  - about reducing risk
  - about how to comply with government directives
- What could make social distancing at work easier? [priorities]

**Impact**

***So now, thinking about the situation with COVID 19 and the directives and restrictions put in place to control the pandemic what impact are these having*** *[to be adapted for type of employer and business e.g. care, customer, supply]* ***on the organisation?***

***[Perhaps I could ask you to think first about how things were at the start of lockdown and then more recently as the government moves towards relaxing some restrictions]***

*Additional prompts*

- On the way the service operates
- Service delivery
- Staff/clients etc
- Concerns for self/staff related to current directives
- Concerns for the communities served of current directives or the effect of the virus itself
- Changing concerns during phases of lockdown

*How are members of the public/customers/clients currently being involved in service related decisions during the pandemic*? [e.g. consultation/online community/resident committees]

***Is there anything you would you like to see change currently?***

- - What? Why? How?

*Additional prompt:*

- - - Anything that would help or hinder these changes
    - Within the organisation
    - Within households and communities
    - At a political or strategic level
- ***What concerns do you have for the aftermath of the pandemic?***
- ***Does the organisation have any plans for the aftermath of the pandemic?***

*Prompts:*

- - Priorities for recovery period
  - Needs they predict will need to be addressed and how

Finally, is there anything that I haven’t asked about that you would like to add before we end the interview?

Thank you for participating.
